# Oil Phase Solubility Rather Than Diffusivity Determines the Release of Entrapped Amino Acids and Di-Peptides from Water-in-Oil-in-Water Emulsions

**DOI:** 10.3390/molecules27020394

**Published:** 2022-01-08

**Authors:** Esra Kocaman, Davide Rabiti, Juan Sebastian Murillo Moreno, Asli Can Karaca, Paul Van der Meeren

**Affiliations:** 1 Department of Food Engineering, Faculty of Chemical and Metallurgical Engineering, Istanbul Technical University, Maslak 34469, Turkey; cankaraca@itu.edu.tr; 2 Particle and Interfacial Technology Group, Faculty of Bioscience Engineering, Ghent University, Coupure Links 653, B-9000 Ghent, Belgium; Davide.Rabiti@ugent.be (D.R.); JuanSebastian.MurilloMoreno@ugent.be (J.S.M.M.); Paul.VanderMeeren@UGent.be (P.V.d.M.)

**Keywords:** double emulsion, encapsulation, hydrophobicity, di-peptides, amino acids, diffusivity

## Abstract

The permeation of amino acids and di-peptides with different hydrophobicities across the oil phase in W/O/W double emulsions was investigated at different concentrations, considering the pH of the aqueous phase. Moreover, the particle size, yield of entrapped water and release kinetics of the double emulsions was evaluated as a function of time. Regarding the release of the entrapped amino acids and di-peptides, their hydrophobicity and the pH had a significant effect, whereas the concentration of the dissolved compound did not lead to different release kinetics. The release of the amino acids and di-peptides was faster at neutral pH as compared to acidic pH values due to the increased solute solubility in the oil phase for more hydrophobic molecules at neutral pH. Regarding the effect of the type of oil, much faster amino acid transport was observed through MCT oil as compared to LCT oil, which might be due to its higher solubility and/or higher diffusivity. As di-peptides released faster than amino acids, it follows that the increased solubility overruled the effect from the decreased diffusion coefficient of the dissolved compound in the oil phase.

## 1. Introduction

Water-in-oil-in-water (W1/O/W2) emulsions allow to protect encapsulated water-soluble functional ingredients in the internal water phase and enable controlled release of these compounds, e.g., in the gastrointestinal system [1]. The double emulsion encapsulation system was found to be efficient in preventing degradation of several substances in the gastro-intestinal track, such as betalain [2], vitamin B12 [3], caffeine [4], peptides [5] insulin [6], antioxidants [7], iron [8] and plant bioactives [9].The encapsulated ingredients have, however, a high tendency to diffuse from the internal water phase to the external water phase [10].

Most small neutral and drug molecules are transported passively across the membrane. Passive transport is a type of transport in which solutes move along their respective concentration gradients, which means that the solutes tend to migrate from a zone of higher concentration to a zone of lower concentration [11]. Passive diffusion largely depends on the physicochemical properties of the double emulsion system, as well as of the encapsulated functional ingredient, such as hydrophobicity, polarity and molecular size [12].

The influence of the droplet size of the emulsions, the osmotic pressure of the internal and external water phases, as well as the type and concentration of the hydrophobic and hydrophilic emulsifiers on the stability of double emulsions has been extensively studied [13,14]. Moreover, the effect of the double emulsion characteristics on the encapsulation and release of functional ingredients has been studied. The large effect of the (unadsorbed) PGPR concentration was pointed by some authors [8,15], whereas other studies [16,17] described the effect of the interfacial composition, which may be further controlled by cross-linking. However, as far as we know, there are no studies related to the effect of the molecular properties of the entrapped compounds on the stability of double emulsions, as well as on their encapsulation and release properties.

In order to enable a systematic study of the effect of the molecular properties of the functional component, we selected amino acids and simple peptides. It is indeed well known that the 20 naturally occurring amino acids can be subdivided into hydrophobic and hydrophilic amino acids, which leads to the specific folded structure of globular proteins in aqueous media. Moreover, amino acids, the subunits of peptides, play a critical role in the metabolism and neurotransmission, whereas peptides act as hormones, growth factors and antimicrobials. The physiological benefits of these compounds, such as their anti-inflammatory, anticancer, antimicrobial and antioxidant activities, make them good therapeutic drug candidates in treating pain, cancer and neurological diseases [18]. In recent years, bioactive peptides have shown many health-promoting effects to be used in healthy food formulations of functional foods and nutraceuticals. However, the acidic environment of the gastrointestinal system may lead to the degradation of amino acids and peptides, which makes the protection of these compounds necessary. 

The current contribution is an extension to our previous work, in which we started to explore the influence of hydrophobicity on the release of amino acids. From our previous research on the release kinetics of different amino acids from the internal aqueous phase of double emulsions, leucine was found to be much more rapidly released as compared to more hydrophilic amino acids, such as glycine and glutamine, whereas valine and alanine had an intermediate behaviour [19]. This was explained by the increased solute solubility in the intermediate oil phase as the hydrophobicity of the amino acid increased. However, the solution–diffusion transport model suggests that the release kinetics of solutes do not only depend on their hydrophobicity, but also on their diffusivity, which is inversely proportional to molecular size. Hence, in this study, the encapsulation and release properties of amino acids and di-peptides were compared. Moreover, the effect of the aqueous phase pH was investigated as this characteristic is known to affect the degree of ionization of amino acids and peptides, and hence their hydrophobicity. Hereby, pH values ranging from 1.0 to 7.0 were considered, as this pH range is encountered within the gastro-intestinal tract. Last but not least, the effect of the oil phase composition was evaluated as this factor will also affect the distribution of the amino acids and di-peptides between the oil and the aqueous phases. Overall, the main goal of this study is to contribute to a more in-depth knowledge of the factors driving the encapsulation and release of water-soluble compounds, which is of vital importance in various applications including pharmaceuticals, cosmetics and functional foods [13]. In the latter case, encapsulation of water-soluble compounds in the internal aqueous phase may have a number of advantages, such as their protection from harsh conditions (such as a low pH in the stomach), the avoidance of a bad taste perception upon oral ingestion, or their controlled release (which may help to increase satiety and hence reduce oral food intake). 

## 2. Results and Discussion

In the current research, the release of amino acids and di-peptides from W1/O/W2 emulsions has been evaluated. Moreover, the concentration of the entrapped compounds and the pH of the aqueous phases were considered as variables. Hereby, the release of amino acids was investigated from double emulsions based on either long chain triglycerides (LCT) or medium chain triglycerides (MCT) since the oil phase composition is known to largely affect the solubility of the entrapped compounds. To characterize the double emulsion stability, the average droplet size and entrapped water volume fraction have been measured during storage.

### 2.1. Double Emulsion Characterization

#### 2.1.1. Determination of the Average Droplet Size

**Figure 1:**
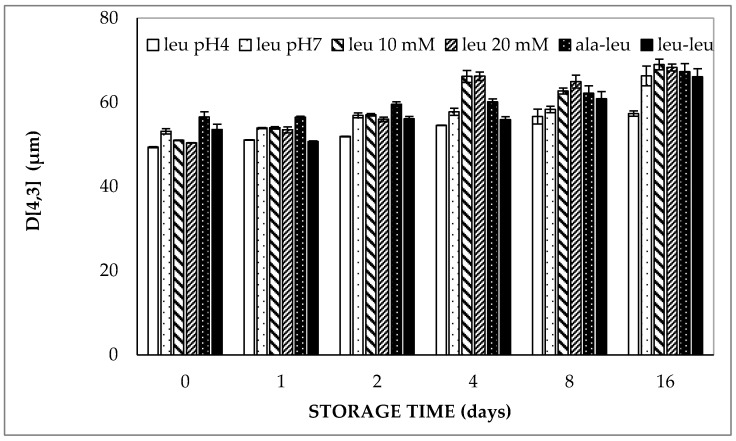
Volume-weighted average droplet diameter (D[4,3]) of double emulsions containing either 5 mmol/L (at pH 4.0 or pH 7.0), 10 mmol/L or 20 mmol/L L-leucine, 5 mmol/L alanine–leucine or 5 mmol/L leucine–leucine (all at pH 7.0) prepared with HOSO upon storage at 37 °C.

Regarding the results of the double emulsions containing LCT oil, the volume weighted average droplet size was about 50 µm directly after preparation and increased up to about 60–65 µm at 37 °C within 16 days (Figure 1). The significant increase in volume weighted average droplet size (D[4,3]) during 16 days was confirmed for all double emulsions containing high oleic sunflower oil (HOSO) by regression analysis (*p* < 0.05). It should be noted that the volume-weighted average diameter of the different samples considered in Figure 1 varied from 49.4 to 56.5 µm directly after preparation. Considering that these samples were independently prepared and hence might have experienced small differences in homogenization intensity, it follows that the effect of the enclosed hydrophilic compound on the particle size of the different double emulsions was limited. This is especially true, considering the fact that the volume-weighted average droplet diameter of two independently prepared double emulsions containing 5 mmol/L L-leucine (at pH 7.0) was 50.2 and 53.1 µm, respectively, directly after preparation. 

Linear regression of the volume-weighted average droplet diameter versus storage time clearly indicated that no significant differences could be found in the droplet size increase in the double emulsions over time, regardless of the pH of the aqueous phases, concentration and molecular size of the entrapped compound (Table 1): all slopes ranged from about 0.47 ± 0.34 (for 5 mmol/L L-leucine at pH 4.0) to 1.03 ± 0.98 µm/day (for 20 mmol/L L-leucine at pH 7.0). On the other hand, all slopes were significantly larger than zero, indicating that the sizes significantly increased during storage. The latter observation is in line with our previous study in which it was shown that the size increase during storage was due to flocculation, rather than coalescence [19].

On the other hand, the volume-weighted average droplet size of the double emulsion containing MCT oil was about 34 µm immediately after preparation and reached about 50 µm after 16 days of storage at 37 °C. The daily increase in average droplet size of the double emulsion with MCT oil was 0.64 ± 0.72 µm/day (r^2^ = 0.60), which shows that the average droplet size of the double emulsion with MCT did not significantly increase during 16 days of storage (*p* > 0.05). From the Tukey post hoc analysis, the double emulsion prepared with MCT oil had a significantly smaller average droplet size compared to those containing LCT oil (*p* < 0.05). The smaller oil droplet size of the MCT-containing double emulsion can be explained by the lower viscosity of MCT oil, giving rise to a viscosity ratio between continuous and dispersed phase closer to one [20].

#### 2.1.2. Yield of Entrapped Water

**Figure 2:**
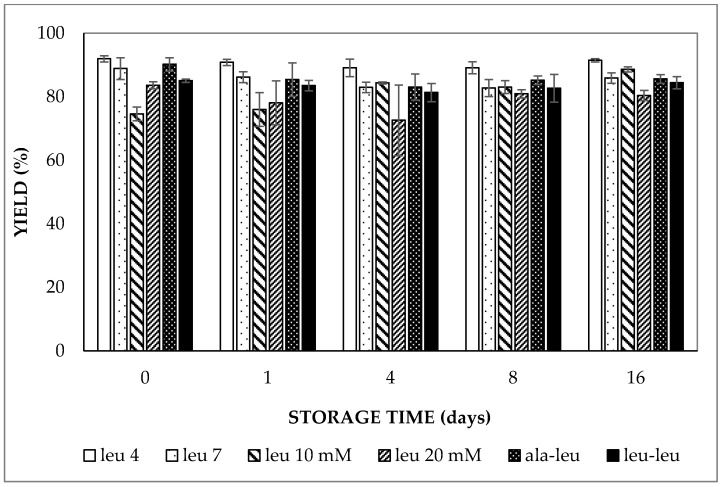
Yield of entrapped water in double emulsions containing either 5 mmol/L (at pH 4.0 or pH 7.0), 10 mmol/L or 20 mmol/L L-leucine, 5 mmol/L alanine–leucine or 5 mmol/L leucine–leucine (all at pH 7) prepared with HOSO upon storage at 37 °C.

From the analytical photocentrifugation results, the entrapped water volume fraction was found to be 80–90% of the theoretical value (based on the relative contribution of the internal water phase to the combined internal and external water phases) for all double emulsions prepared with LCT oil (Figure 2); the latter is also indicated as the yield. The entrapped water volume fraction of the double emulsions did not show a significant increase or decrease within 16 days of storage regardless of the pH of the aqueous phases, the concentration and the molecular size of the entrapped compound (*p* > 0.05). Similarly, the entrapped water volume fraction fluctuated around 90% for the double emulsion containing MCT oil during 16 days of storage. This constant yield is a logical consequence of the similar osmotic pressure of the inner and outer water phases and also indicates that no entrapped water droplets (and entrapped solute) were lost by external coalescence during storage.

### 2.2. Release Profiles of the Entrapped Compounds

#### 2.2.1. Influence of L-Leucine Concentration on Release Kinetics

In our previous research, the encapsulation and release of amino acids (such as leucine) at a concentration of 5 mmol/L was considered [19]. For functional food applications, the required dose is inversely proportional to the concentration of the functional ingredient in the double emulsion. Hence, the effect of increasing amino acid concentrations (from 5 to 40 mmol/L) was investigated in order to minimize the required dose. 

**Figure 3:**
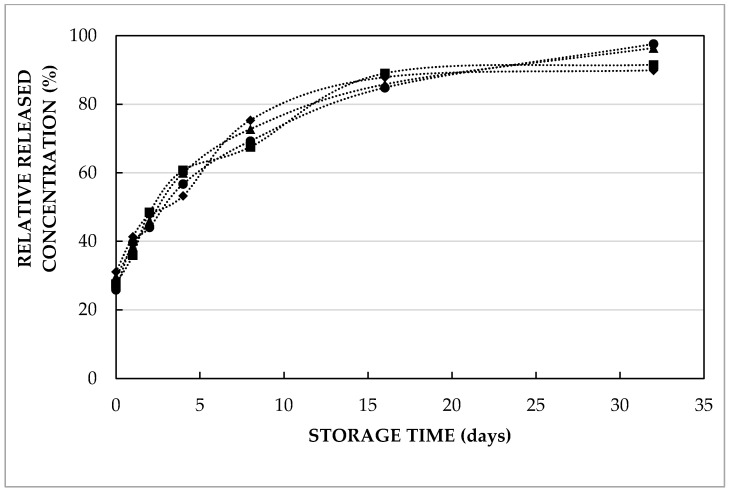
Relative released L-leucine concentration (i.e., actual concentration relative to the expected concentration upon homogeneous distribution over the combined aqueous phases) in the external phase as a function of storage time at 4 °C of double emulsions containing 5 mmol/L (diamonds), 10 mmol/L (circles), 20 mmol/L (triangles) or 40 mmol/L (squares) leucine prepared with HOSO at pH 7.

Both Figure 3 and Table 2 indicate that double emulsions containing varying concentrations of L-leucine showed a similar trend to be released to the outer aqueous phase during 32 days of storage at 4 and 37 °C. The only exception was the double emulsion containing 20 mmol/L, which was significantly different from the one containing 5 mmol/L (*p* = 0.04) and 10 mmol/L (*p* = 0.02) at 37 °C. From Figure 3, the equilibrium concentration in the released amino acid was observed after approximately 2 weeks of storage at 4 °C. Table 2 represents the average residence time (t_a_) and initial amino acid concentration in the external aqueous phase (C_0_) of double emulsions containing different concentrations and different types of entrapped compounds in the inner water phase at varying pH during storage at 37 °C. Due to the release of amino acids and di-peptides in the second emulsification step, the initial concentration (represented by C_0_) differed from 0. However, the release during the preparation was limited: the released L-leucine concentration just after preparation was only about 10–15%, irrespective of the entrapped L-leucine concentration.

For completeness, it should be mentioned that the estimated t_a_ values, i.e. the time until 63% of the entrapped compound releases, for L-leucine release at 4 °C were 6.87 ± 1.09, 8.63 ± 1.23, 7.46 ± 0.70 and 7.79 ± 2.47 days, for double emulsions containing 5, 10, 20 and 40 mmol/L L-leucine, respectively. This shows that the release kinetics were not affected by the entrapped solute content. These results show that the entrapped solute concentration in the double emulsion formulation can be increased (within the concentration range studied), and hence that the required volume of double emulsion to obtain a predefined amount of entrapped functional compound can be reduced without any negative impact on the functionality (in terms of release kinetics). Looking at the estimated kinetic constant (t_a_) parameter values at 4 or 37 °C (Table 2), it is obvious that the release at 4 °C was roughly 6–10 times slower as compared to 37 °C. The faster release of L-leucine at higher temperature was explained by both increased solubility and diffusivity in our previous study [19]. From a practical point of view, this is an interesting observation, as this temperature dependency ensures a much slower release during (refrigerated) storage, whereas faster release is induced upon ingestion and digestion (at 37 °C).

#### 2.2.2. Influence of Oil Phase Composition

**Figure 4:**
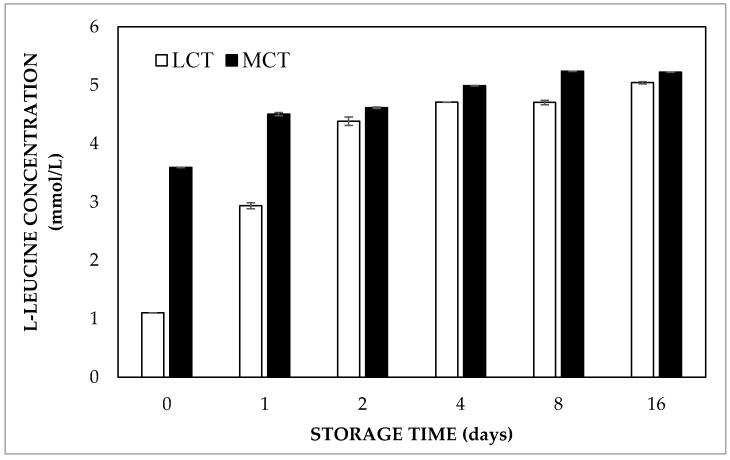
Released concentrations of L-leucine in double emulsions containing either MCT or LCT oil at 37 °C.

In order to investigate the oil phase composition effect, LCT and MCT oil were used in double emulsions. It was noted that the L-leucine concentration in the external aqueous phase directly after preparation was almost 3.60 mmol/L in double emulsions prepared with MCT oil, whereas it was only about 1 mmol/L for LCT containing samples (Figure 4). Moreover, the equilibrium concentration was observed within 4 days of storage for the double emulsions prepared with MCT oil, while it took about 16 days for the samples containing LCT oil. As the entrapped water volume fraction of the double emulsions containing HOSO or MCT oil was found to be 91.8% and 92.9% just after preparation, respectively, it is clear that L-leucine release did not occur via breakdown of the internal water droplets. The faster release of L-leucine was likely due to the fact that MCT oil is more hydrophilic than LCT, as reflected by the solubility of water in the oils, which is more than two times higher in MCT as compared to LCT [21]. Hence, faster transport of L-leucine through the oil phase occurred due to the higher solubility of the solute. In addition, MCT oil also has a lower viscosity, which gives rise to a higher diffusivity of dissolved solutes, and hence may also speed up molecular transport. With respect to the oil phase composition, it was also observed that highly hydrophobic mineral oil induced a slower release of peptides during gastric digestion compared to butter oil and linseed oil [22]. This was explained by the higher interfacial tension and viscosity that mineral oil provided to the double emulsions.

#### 2.2.3. Influence of Molecular Properties on Release Kinetics

**Figure 5:**
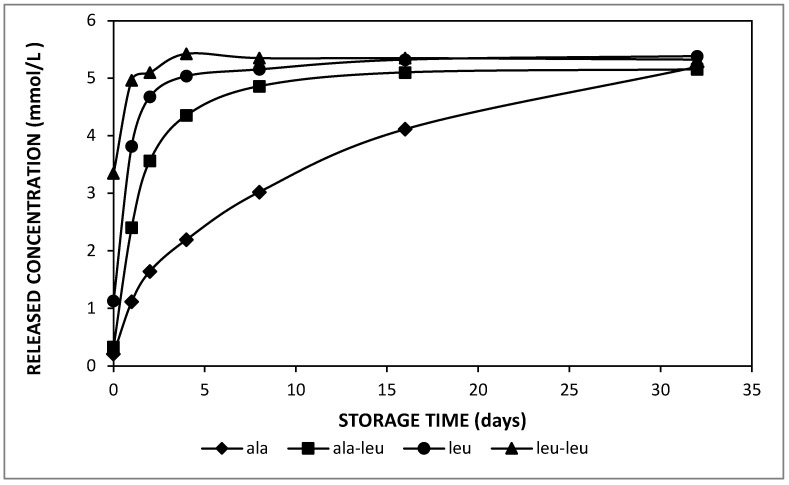
Released concentration in the external phase of double emulsions containing alanine (diamonds), L-alanine–L-leucine (squares), leucine (circles) and L-leucine–L-leucine (triangles) prepared with HOSO at pH 7 during storage at 37 °C.

In order to enable to discriminate between the effect of solubility and diffusivity, which are directly proportional when considering the effect of temperature or the effect of the oil phase composition on the amino acid release, the release properties of amino acids versus di-peptides were considered: as di-peptides have a larger molecular weight and hence bigger molecular dimensions, as well as a higher hydrophobicity, it follows that the solubility and diffusivity are inversely related when considering amino acids versus di-peptides. Concerning the molecular properties of the entrapped compounds on the release kinetics, L-leucine–L-leucine indicated the most significant trend to be released to the external phase of the double emulsions, whereas alanine was released slowest during 32 days of storage at 37 °C (Figure 5). Table 3 indicates that L-leucine–L-leucine was the most hydrophobic compound entrapped in the current study.

It also had the lowest average residence time (t_a_) among all entrapped solutes and thus released fastest at 37 °C. The slowest release was observed in the double emulsion containing DL-alanine. From Table 3, these data clearly show that the solute solubility in the oil phase (which is proportional to its hydrophobicity) is much more decisive than the solute diffusivity; as the latter is inversely proportional to molecular size, the largest value is expected for alanine and the smallest for L-leucine–L-leucine. Moreover, the estimated equilibrium concentration (C_eq_) in the external phase was close to the expected value for all emulsions. The amino acid or dipeptide concentration approached an equilibrium after only 1 day for L-leucine and L-leucine–L-leucine, whereas it took about 16 days for L-alanine-L-leucine at 37 °C. It was also observed that the initial amino acid concentration in the outer water phase was close to 0 for the double emulsion that contained DL-alanine, which shows the very limited release during preparation. On the other hand, the initial concentration for the other solutes increased as their average residence time (t_a_) was decreased, which points to the fact that this initial release was also due to a solution–diffusion mechanism during emulsification.

In the present work, it is clearly seen that the release of entrapped compounds significantly depends on the hydrophobicity of the enclosed solute. Hereby, the permeability of small molecules across the intermediate oil phase separating the two aqueous phases can be explained by the solution–diffusion model.

According to Overton’s rule, the lipid membrane permeability of a molecule increases with its hydrophobicity. Our data indicate that the same holds for the permeability of an oil phase. A similar observation was reported that the rate of transfer across the oil layer in a W/O/W emulsion of the hydrophobic L-tryptophan was greater than the rate of the hydrophilic vitamin B [26]. However, in another study, the rate of release in W/O/W was found to be higher for hydrophilic catechin as compared to hydrophobic curcumin in the gastrointestinal environment, which was claimed to be due to the fact that curcumin tends to remain within the lipid phase, whereas catechin diffuses readily to the hydrophilic releasing media [27].

Concerning the peptide permeation, it was found that an increase in the hydrophobicity of di-peptides led to an enhanced interaction with phospholipid membranes, whereas less hydrophobic di-peptides were expelled from the surface [28]. It was also mentioned that the hydrophilic serine–serine dipeptide desorbed from the interface to the aqueous phase, whereas hydrophobic phenylalanine–leucine and amphiphilic serine–leucine tended to accumulate at the interface. These findings clearly prove that the molecular structure of the entrapped compounds critically influences their release kinetics [29].

According to the solution–diffusion transport model, the release kinetics of entrapped hydrophilic compounds in double emulsions depend on both the solute solubility and diffusivity in the oil phase. For the hydrophobicity effect, the compounds with higher hydrophobicity will be more soluble in the oil phase, and thus they will release faster. Considering the data of Figure 5, it is clear that the solubility effect largely overrules the diffusivity as the release kinetics are proportional to the molecular weight, i.e., slower for smaller molecules, whereas the opposite effect should be observed if diffusivity played an important role.

#### 2.2.4. Influence of pH on Release Kinetics

As the solubility and partitioning of hydrophilic molecules with ionisable functional groups, such as carboxyl and amino groups, largely depend on the environmental pH, the release of entrapped solutes was examined at neutral as well as acidic pH conditions [30]. Whereas the former conditions are typically found in the intestines, the latter relate to gastric conditions.

**Figure 6:**
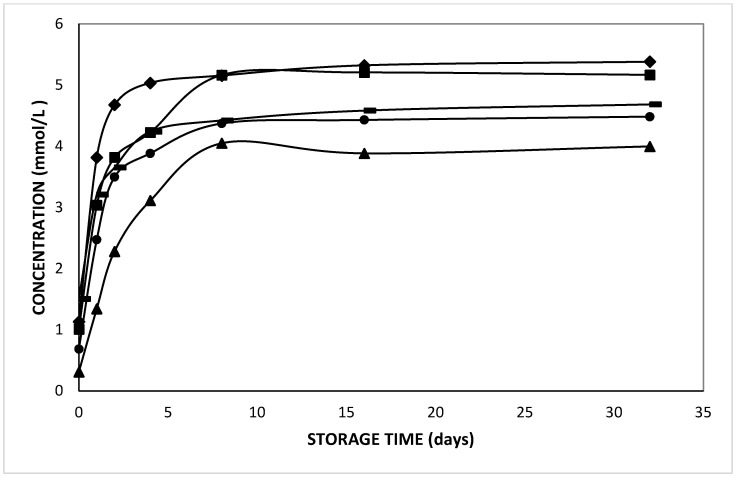
Released L-leucine concentration in the external phase of double emulsions containing 5 mmol/L L-leucine at pH 1 (triangles), pH 2 (circles), pH 3 (dashes), pH 4 (squares), and pH 7 (diamonds) prepared with HOSO during storage at 37 °C.

In the current study, the release of L-leucine at different pH values from double emulsions was found to be significantly different; the only exception was the double emulsion at pH 3 which was not significantly different from the one at pH 4 (*p* = 0.29). Figure 6 indicates the pH effect on the release of L-leucine during 32 days of storage at 37 °C. It was observed that the release rate of L-leucine was lowest at pH 1 while it was highest at pH 7. Moreover, a remarkable difference in L-leucine release was observed during preparation, which was at least 1.25 mmol/L at pH 7, whereas it was only 0.26 mmol/L at pH 1. Hence, the initial released L-leucine concentration increased with increased pH of the aqueous phases.

Furthermore, from Table 2, it is clear that the average residence time (t_a_) of L-leucine increased as the pH of the aqueous phases decreased, which indicates a slower release for the positively charged species formed at acidic pH. The net charge of amino acids and peptides is pH dependent. From Table 3, the isoelectric point of the compounds can be observed. Looking at the pK_a_ value, more than 99% of L-leucine was in the zwitterionic state at pH 7. At lower pH, the amino acids and di-peptides became positively charged. The Henderson–Hasselbalch equation revealed that 95, 67, 17 and 2% of cationic L-leucine was present at pH 1, 2, 3, and 4, respectively. Hereby, the cationic species are less permeable than the zwitterionic form due to their lower hydrophobicity. Hence, we observed that the release of neutral and weakly charged (non-polar) solutes was promoted by their higher solubility in the oil phase, as compared to (polar) charged amino acids and di-peptides.

It should be also noted that a lower equilibrium concentration was observed for the double emulsions with lower pH values (Figure 6). Upon pH adjustment to acidic values, the solutes in the internal water phase become highly protonated, and hence cationic. As the released species from the internal to the external water phase were thought to be rather zwitterionic, they will become protonated in the external phase, which will cause an increased pH in the external water phase. Conversely, the release of the zwitterionic species will induce a partial deprotonation of the retained cationic solute. This effect will lower the pH in the internal water phase, which facilitates a further protonation (Figure 6). This effect will ultimately stop the further efflux of (cationic) entrapped solute, despite a concentration gradient between the internal and external aqueous phases. Hence, the positively charged solutes will be partly retained inside the internal water droplets.

For the case of double emulsions containing L-leucine–L-leucine, the release profile at different pH values was significantly different, except for the double emulsion at pH 7 which was not different from pH 2 (0.11) and pH 3 (*p* = 0.31). When the t_a_ values of the double emulsions containing L-leucine–L-leucine were considered, the highest residence time was found for the double emulsion at pH 2 and the lowest at pH 7 (Table 2).

Some studies regarding the release of compounds as a function of pH are in agreement with the current work. Thus, it was reported that the rate of tryptophan release was higher at pH values near the isoelectric point whereas ionized forms released slower due to their lower solubility in oil [26]. Additionally, the release of peptides in the gastrointestinal environment in the absence of lipase was investigated and it was found that the release is controlled by the peptide hydrophobicity: peptides with a higher hydrophobicity index showed a higher release rate [22]. Moreover, these authors also found higher release rates at intestinal conditions (i.e., at pH 7) than at gastric pH (at pH 3) since neutral and weakly charged (non-polar) peptides are more soluble in oil as compared to charged peptides. In general, the colloidal system containing the bioactive compounds experiences a complex series of physicochemical and physiological processes during digestion as it passes through the different regions. Firstly, the delivery system is exposed to changes (i.e., dissolution, dilution, and dispersion) in the mouth where it is mixed with saliva for about 1 min (at pH 5–7). Secondly, the ingested delivery system is exposed to a high ionic strength and strong acids (at pH 1–3) for about 30 min to 4 h in the stomach, which may affect the charge characteristics of ionizable groups, and hence the release properties of bioactives. The latter highly depend on the type of encapsulated compound: whereas zwitterionic amino acids and peptides are less permeable at low pH, the opposite will hold for weakly acidic compounds, such as ferulic acid. Upon leaving the stomach, the colloidal system will be further processed in the small intestine for 1–2 h at neutral pH conditions: thereby, the pH gradually increases from about pH 6 in the duodenum to about pH 7.4 in the terminal ileum [31]. Hence, different pH conditions prevail in different parts of the gastro-intestinal tract, which opens interesting perspectives for targeted delivery of ionisable functional compounds.

However, in comparing our results to those obtained during actual digestion in the gastrointestinal track, it is important to realize that the delivery system in the latter case is not only exposed to pH variations, but also to the effect of enzymes, ionic strength variations, mechanical forces, and additional compounds in the gastric and intestinal fluids (such as bile salts) [31]. Some authors examined the release of bioactive compounds from double emulsions in the presence of enzymes in simulated gastrointestinal track conditions [13,32,33]. In the presence of digestive enzymes (i.e., pepsin, lipase), hydrolysis will significantly affect the release mechanism as triglyceride hydrolysis of the oil which forms the intermediate phase in the double emulsions disrupts the protective barrier between both aqueous phases, and hence accelerates the release rate of entrapped compounds as a result of thinning of the lipid barrier [2]. Hence, the release kinetics of enclosed functional compounds is a complex multi-factor phenomenon.

## 3. Materials and Methods

### 3.1. Materials

The amino acids and di-peptides encapsulated for this study were DL-alanine, L-leucine (Acros Organics, Geel, Belgium), L-alanine-L-leucine (Sigma-Aldrich, St. Louis, MO, USA) and L-leucine-L-leucine (Sigma-Aldrich, St. Louis, MO, USA). The physicochemical characteristics of the amino acids and di-peptides used in the current study are demonstrated in Table 3. For the oil phase of the emulsions, high oleic sunflower oil (HOSO; Contined B.V., Bennekom, The Netherlands) was used as LCT oil, whereas Miglyol 812N with 58% C8:0 and 41% C10:0 (IMCD, Mechelen, Belgium) was selected as MCT oil. Polyglycerol polyricinoleate (PGPR 4150) was a kind gift of Palsgaard A/S (Juelsminde, Denmark) to be used as the hydrophobic emulsifier. Furthermore, polysorbate 80 was used as the hydrophilic emulsifier (Tween^®^ 80; Sigma-Aldrich, St. Louis, MO, USA). Additional reagents used are potassium chloride (AnalaR NORMAPUR, VWR Chemicals, Leuven, Belgium) as an electrolyte to balance the osmotic pressure between the water phases, and sodium azide (NaN_3_; Sigma-Aldrich, Steinheim, Germany) as an antimicrobial agent.In the colorimetric method for amino acid and dipeptide determination, picrylsulfonic acid solution 5% *w/v* in H_2_O (Sigma-Aldrich, St. Louis, MO, USA), sodium hydrogen carbonate (NaHCO3; AnalaR NORMAPUR^®^, VWR Chemicals, Leuven, Belgium) and hydrochloric acid 32% (HCl; VWR Chemicals, Fontenay-sous-Bois, France) were used.

### 3.2. Methods

To compare the outcomes of this study with our previous research, similar double emulsion preparation and characterization methods have been used [19].

#### 3.2.1. Emulsion Preparation

##### W/O Emulsion Preparation

Equal masses of an aqueous phase containing 100 mmol/L KCl as well as a given concentration of amino acid or dipeptide were added gradually to an oil phase (containing 5% of PGPR) at 24,000 rpm using an Ultra-Turrax (S25-10G, IKA-Werke, Staufen, Germany) during 5 min at 60 °C. Unless stated differently, the internal aqueous phase contained 15 mmol/L of solute at pH 7.0. The pH of the internal and external water phases was adjusted to the desired values using 0.5 and 1 mol/L HCl and NaOH.

##### W/O/W Emulsion Preparation

Equal masses of external water phase and W/O emulsion were mixed using an Ultra-Turrax S25-10G (IKA^®^Werke, Staufen, Germany) at 17,500 rpm for 5 min. To ensure isotonic conditions, the KCl concentration in the external phase was equal to that in the internal phase (i.e. 100 mmol/L) plus half of the amino acid or peptide concentration in the internal phase. Hence, 0.1075 mol/L KCl was used when 15 mmol/L solute was added in the internal aqueous phase.

Considering the 25/25/50 mass ratio of the W/O/W, the equilibrium concentration (i.e., the concentration reached upon homogeneous distribution of the solute over the internal and external aqueous phases) was expected to be one third of the concentration that was used in the internal water phase (W1). Unless stated differently, the reported solute concentrations in the remainder of the manuscript refer to these averaged concentrations over the combined aqueous phases.

#### 3.2.2. W/O/W Emulsion Characterization

The yield of entrapped water (using analytical centrifugation) as well as the volume weighted average diameter (D[4,3]; using laser diffraction) of the double emulsion droplets were measured as described before [19]. In the latter case, the samples were diluted using a solution with the same KCl concentration as the W2 phase in order to keep the osmotic balance.

#### 3.2.3. Determination of Released Amino Acid/Dipeptide Release Kinetics

The collection of the external water phase samples and spectrophotometric determination of the amino acid and dipeptide concentration was performed according to our previous method with slight modifications [19]. Hereby, the double emulsions were centrifuged (Sigma 1-15P, SIGMA Laborzentrifugen, Osterode am Harz, Germany) at 10,000× *g* for 5 min. The serum phase was extracted using a syringe, then filtered (pore size: 0.25 µm with nylon membrane; VWR International, USA) and stored at 4 °C until analysis. The samples were diluted 10, 20, 40 or 80 times for the initial concentrations of 15, 30, 60 and 120 mmol/L entrapped compounds, respectively. The spectrophotometric determination of the amino acid and di-peptide concentration was performed by mixing 1 mL of diluted sample with 1 mL of a 0.48 mmol/L NaHCO_3_ solution and 0.6 mmol/L TNBS. This mixture was kept in a dark place at 40 °C for 3 h; 1 mL of 1 mol/L HCl was added to stop the reaction. The absorbance of the samples was measured at 340 nm in a spectrophotometer (UV-1600PC UV-VIS, VWR International, Radnor, PA, USA).

The average residence time t_a_ was estimated by fitting a mathematical model to the experimental release data of amino acids and di-peptides as a function of time (t). The initial, released and equilibrium concentrations in the external water phase are indicated by C_0_, *C* and *C_eq_* (in mmol/L), respectively.

C = C_eq_ − (C_eq_ − C_0_).exp(−t/t_a_) (1)

#### 3.2.4. Statistical Analysis

Linear regression analysis was used to analyze the changes in the droplet size and yield of double emulsions. Statistical analysis was performed considering the 95% confidence intervals to check significant differences between samples. Additionally, a Tukey post hoc test, at a confidence value of 95%, was performed in SPSS to check the significance of the differences between the yields of double emulsions. The release of amino acids and dipeptides was analyzed in SPSS using a paired *t*-test or Wilcoxon test considering the distribution.

## 4. Conclusions

The results of this study suggest that the release of functional ingredients (such as amino acids and di-peptides) from the internal aqueous phase of double emulsions is controlled by the molecular properties of the entrapped compound (with hydrophobicity overruling molecular size), by the oil phase composition and by the pH of the aqueous phases. Amino acid transport was slower through the more hydrophobic LCT-oil as compared to MCT-oil. The concentration of the entrapped compound, on the other hand, did not change the release rate. The latter observation is of great practical importance, as a higher concentration of the compounds enables the use of a smaller dosage for the same effect. A faster release of the amino acids and di-peptides was found at neutral pH as compared to gastric pH conditions, which was due to the higher solubility of zwitterionic compounds in oil as compared to (more polar) charged solutes. This opens interesting perspectives for controlled release, as early release in the stomach is prevented by its low pH. From a practical point of view, our results may provide guidance in the design of colloidal systems for the encapsulation and sustained release of ingredients for functional food applications by further exploring the impact of the molecular properties of the ingredient (such as hydrophobicity and diffusivity), as well as the impact of environmental conditions (such as pH and oil phase polarity). As the double emulsions in this study typically contained oil droplets larger than 10 µm, they are preferentially applied in water-continuous functional foods, drinks or nutraceuticals with a high viscosity (to prevent creaming) that do not have to be optically clear, such as yoghurt, mayonnaise, salad dressings, or other structured foods.

## Figures and Tables

**Table 1:** Average rate of change (and 95% confidence interval) and determination coefficient (r^2^) as determined by linear regression analysis of the volume-weighted average droplet size during 16 days of storage at 37 °C (expressed in µm/day) of double emulsions containing HOSO.

Solute	pH	Average Solute Concentration (mmol/L)	r^2^ (-)	Slope (µm/day)
Ala	7.0	5	0.84	0.95 ± 0.58 ^a^
Leu	1.0	5	0.97	0.60 ± 0.16 ^a^
	2.0		0.83	0.65 ± 0.41 ^a^
	3.0		0.89	0.52 ± 0.25 ^a^
	4.0		0.79	0.47 ± 0.34 ^a^
	7.0		0.94	0.75 ± 0.27 ^a^
		10	0.70	0.99 ± 0.89 ^a^
		20	0.67	1.03 ± 0.98 ^a^
		40	0.69	0.72 ± 0.66 ^a^
Ala-Leu	7.0	5	0.96	0.66 ± 0.19 ^a^
Leu-Leu	7.0	5	0.91	0.87 ± 0.38 ^a^

^a^ no significant differences were observed (with 95% confidence).

**Table 2:** Estimated average residence time (t_a_) and initial concentration in the external aqueous phase (C_0_) (with 95% confidence interval) of amino acids and di-peptides in double emulsions prepared with HOSO as a function of the average solute concentration in the aqueous phase and the aqueous phase pH, during storage at 37 °C.

Entrapped Compound	Average Solute Concentration (mmol/L)	pH	t_a_ (d)	C_0_ (mmol/L)
Ala	5	7.0	7.50 ± 2.30	0.50 ± 0.17
Ala-Leu			1.80 ± 0.07	0.37 ± 0.08
Leu	5		1.27 ± 0.21	1.14 ± 0.06
	10		1.35 ± 0.12	1.55 ± 0.30
	20		1.22 ± 0.18	2.54 ± 0.92
	40		0.80 ± 0.15	5.20 ± 2.03
	5	1.0	2.67 ± 0.11	0.26 ± 0.16
		2.0	1.55 ± 0.10	0.71 ± 0.14
		3.0	1.47 ± 0.10	1.56 ± 0.15
		4.0	1.86 ± 0.28	1.13 ± 0.26
		7.0	1.27 ± 0.21	1.14 ± 0.06
Leu-Leu		2.0	1.16 ± 0.10	1.41 ± 0.12
		3.0	0.84 ± 0.02	2.38 ± 0.08
		4.0	0.95 ± 0.10	4.29 ± 0.16
		7.0	0.66 ± 0.03	3.35 ± 0.09

**Table 3:** Chemical properties of the amino acids and di-peptides used; the octanol–water partition coefficient (Log Pow) is an indicator of hydrophobicity of the amino acids and di-peptides.

Amino Acid/ Di-Peptide	Formula	MW (Da)	Log Pow	pK_a,1_ [23]	pK_a,2_ [23]	p*I* [23]
Ala	C_3_H_7_NO_2_	89.10	−2.89 [24]	2.34	9.69	6.00
Leu	C_6_H_13_NO_2_	131.18	−1.61 [24]	2.30	9.60	5.98
Ala-Leu	C_9_H_18_N_2_O_3_	202.25	−2.35 [25]	2.34	9.60	5.98
Leu-Leu	C_12_H_24_N_2_O_3_	244.34	−1.46 [25]	2.30	9.60	5.98

## Data Availability

The data that support the findings of this study are available from the corresponding author upon reasonable request.
